# Recent Trends in Microbial Inoculants in Agriculture

**DOI:** 10.1264/jsme2.ME2804rh

**Published:** 2013-12-20

**Authors:** Koki Toyota, Takayoshi Watanabe

**Affiliations:** 1 Graduate School of Bio-Applications and Systems Engineering, Tokyo University of Agriculture and Technology, 2–24–16 Nakacho, Koganei, Tokyo, 184–8588, Japan; 2 Tsukuba Research Institute, Katakura Chikkarin Co., Ltd., 5–5511 Namiki, Tsuchiura, Ibaraki, 300–0061, Japan

Soil microbes have great potential for agriculture. A wide variety of microbes have been utilized to enhance crop production. In the current issue, enhanced growth of rice plants is reported with the inoculation of a nitrogen (N)-fixing bacterium, *Azospirillum* sp. B510 (2), which possesses a disease-suppressive property against the rice blast fungus (*Magnaporthe oryzae*) and the bacterial leaf blight pathogen *Xanthomonas oryzae* (25). The importance of biological nitrogen (N) fixation has been recalled just recently in the N acquisition in tropical rain forest development (5).

The development of mass production and inoculation techniques makes culturable microbes much more attractive research targets among numerous and diverse soil microbes. Beneficial microbial inoculants in agriculture are mainly plant growth-promoting bacteria and fungi, and they function through different mechanisms, *e.g.*, the supply of nutrients, the production of plant hormones, and the suppression of various crop pests.

N is the most limiting nutrient in crop production, on many occasions, and, thus, there are increasing applications of symbiotic or free-living N-fixing bacteria in sustainable agricultural systems. Biomass production of sugarcane and oil palm was increased by the inoculation of *Enterobacter* spp. (12) and *Bacillus sphaericus* (26) strains, respectively, both of which were isolated from the rhizosphere and possessed N-fixing ability. N fixation by the inoculants contributed to increased N nutrition in the host plants. An energetic screening revealed that diverse rhizobacteria possessing N-fixing ability had a plant growth-promoting effect on rice and were a source of biofertilizers (8).

Masunaka *et al.* (14) reported an example in which the solubilization of minerals in the soil is the main effect of a plant growth-promoting fungus, *Trichoderma koningi*. The PGPF does not produce plant hormones (9) and behaves like mycorrhizal fungi in the establishment of symbiotic associations rather than fungal parasites. A lower production of an isoflavonoid, phytoalexin vesitol, a major defensive response of leguminous plants, was involved in their symbiotic associations.

Quorum sensing is a population density-dependent regulation mechanism used by bacteria to regulate gene expression; many Gram-negative plant pathogens control the expression of virulence factors with their quorum-sensing systems (22). *N*-acyl-L-homoserine lactones (AHLs) are signal compounds involved in quorum sensing, and AHL-degrading bacteria have been utilized in the biocontrol of plant diseases (21). *Chryseobacterium* spp. (16) and *Microbacterium* spp. (23) have been isolated from potato roots and leaves, respectively, as AHL-degrading bacteria, and their application as biocontrol agents is expected.

Suppression of plant diseases is an important mechanism in plant growth promotion. Since the reports by Schroth’s group (10, 18), great attention has been paid to colonization of the rhizosphere by fluorescent pseudomonads. More than 800 fluorescent pseudomonad strains were isolated from the phylosphere and rhizosphere of potato plants and characterized for their phylogenic position (20). Some of the strains showed high similarity to *Pseudomonas koreensis* and *P. vancouverensis*, both of which have been reported to be beneficial for biological control or plant growth promotion. For better performance of biocontrol agents against soil-borne pathogens, better colonization on plant roots is essential. Motility and chemotaxis are important traits for root colonization by biocontrol agents. Chemotactic responses of *P. fluorescence* Pf0-1 to amino acids have been studied, and the results revealed that chemotaxis to amino acids, major components of root exudate, and to chemoattractants other than amino acids has an important role in root colonization (15).

Since most plant diseases are caused by phytopathogenic fungi, and most phytopathogenic fungi contain chitin as the main component of their cell walls, chitinolytic microbes are expected to be beneficial for their biological control. A total of 100 chitinolytic bacterial isolates were obtained from the rhizospheres of various agronomic plants in Japan, and their phylogenic positions were revealed (19). Isolates belonging to *Serratia marcescens*, *Stenotrophomonas* spp., and *Lysobacter capsici* have been identified, and their agronomic use is expected.

Plant-parasitic nematodes also cause serious economic damage to many agricultural crops. Effective antagonists against the nematodes have been used as biological control agents. For instance, the genus *Arthrobotrys* is a famous nematode-trapping fungus that has a wide geographical distribution and that captures a live nematode with adhesive trapping organs. Two kinds of such biological control agents are commercially available, Royal 300 and Royal 350 (24), although the fungus traps not only plant-parasitic nematodes but also free-living nematodes that are beneficial in crop production (4). The genera *Paecilomyces* and *Pochonia* are well-known nematode-egg-parasitic fungi that are capable of parasitizing nematode eggs and reducing their populations. *Purpureocillium lilacinum* (=*Paecilomyces lilacinus*) is the main component of BioActs WG^®^ and MeloCon^®^ (1). Barra *et al*. (3) reported that *P. lilacinum* showed virulence against not only plant-parasitic nematodes but also insects, making this agent even more attractive. KlamiC^®^ is a biological control agent containing *Pochonia chlamydosporia* (13). These fungal agents will be used as effective management tools against plant-parasitic nematodes. Bacterial inoculants have also been reported that suppress plant-parasitic nematodes. *L. antibioticus* has the ability to produce lytic enzymes and an antibiotic 4-hydroxyphenylacetic acid, and, in tomatoes, it reduced the severity of the disease caused by the root-knot nematode *Meloidogyne incognita* (11).

Efforts are underway to develop yet-to-be cultivated microbes that will have future applications in agricultural use. Studies of the characterization of culturable microbes are still useful for unraveling their unexploited functions. Eida *et al.* (6, 7) phylogenetically and phenotypically characterized the cellulose-decomposing bacteria and fungi that inhabit composts. Some isolates displayed high cellulose-and hemicellulose-degrading abilities and could be used for improving biodegradation processes during composting, resulting in the production of plant growth-promoting materials.

To meet the demands of an increasing human population, global crop production needs to double, but current estimates are far below what is needed (17). Microbial inoculants should be maximized for different agricultural purposes to enhance crop production and remedy this situation.
